# Supplementary material for: Identification of phenothiazine derivatives as UHM-binding inhibitors of early spliceosome assembly
Source: Nat Commun. 2020 Nov 6;11:5621. doi: 10.1038/s41467-020-19514-1 (PMC7648758; doi:10.1038/s41467-020-19514-1)
Supplement: Supplementary file 1 — Supplementary Information [file 41467_2020_19514_MOESM1_ESM.pdf]

SUPPLEMENTARY INFORMATION

Identification of Phenothiazine Derivatives as UHM-Binding Inhibitors of Early Spliceosome Assembly

Pravin Kumar Ankush Jagtap[1,2,7], Tomáš Kubelka[1,2,7], Komal Soni[1,2], Cindy L Will[3], Divita Garg[1,2], Claudia Sippel[4], Tobias G Kapp[2,5], Harish Kumar Potukuchi[1], Kenji Schorpp[1], Kamyar Hadian[1], Horst Kessler[2,5], Reinhard Lührmann[3], Felix Hausch[4,6], Thorsten Bach[2,8], Michael Sattler[1,2,8]

Contents

Supplementary Table 1 2
Supplementary Table 2 3
Supplementary Figure 1 4
Supplementary Figure 2 5
Supplementary Figure 3 7
Supplementary Figure 4 8
Supplementary Figure 5 9
Supplementary Figure 6 10
Supplementary Methods 11
Supplementary References 24

Supplementary Table 1

**NMR restraints**	
**Distance restraints**	
Total distance restraints	43
Total unambiguous NOE restraints (Intermolecular)	32
Ambiguous interaction restraints (CSP derived)	11
**HADDOCK analysis**	
HADDOCK score	-15.29 ± 11.45
Cluster	1
Cluster size	50
Restraints violation energy	126.3 ± 24.30
Buried Surface area	655.3 ± 36.18
Z-score	0.0
**Violations/quality analysis (dihedral violations)**	
Violations > 5°	0
**CNS energies (kcal/mol) after water refinement**	
Van der Waals energy	-16.2 ± 3.98
Electrostatic energy	-61.8 ± 41.2
Desolvation energy	-3.7 ± 3.1
**RMSD (Å)**	
All backbone residues	0.3 Å
All heavy atoms	0.5 Å
**RMS deviations**	
RMS deviations for bond angles	0.5 °
RMS deviations for bond lengths	0.004 Å
**PROCHECK analysis**	
Residues in most favored region (%)	90.2
Residues in most allowed region (%)	9.8
Residues in most generously allowed region (%)	0
Residues in most disallowed region (%)	0

Supplementary Table 1. Structural statistics for the HADDOCK calculation. Only one cluster with 50 models was obtained by HADDOCK. Structural statistics for the four models with lowest energy are shown are reported here and shown in Supplementary Figure S3.

Supplementary Table 2

Wavelength	1.07 Å
Resolution range	46.87 - 1.94 (2.01 - 1.94)
Space group	P 21 21 2
Unit cell	81.52 89.4 149.21 90 90 90
Total reflections	413362 (40605)
Unique reflections	81205 (7982)
Multiplicity	5.1 (5.1)
Completeness (%)	99.78 (99.84)
Mean I/sigma(I)	12.82 (2.23)
Wilson B-factor	32.00
R-merge	0.076 (0.76)
R-meas	0.085 (0.85)
R-pim	0.037 (0.37)
CC1/2	0.99 (0.72)
CC*	0.99 (0.91)
Reflections used in refinement	81198 (7982)
Reflections used for R-free	4060 (399)
R-work	0.17 (0.25)
R-free	0.20 (0.29)
CC(work)	0.96 (0.85)
CC(free)	0.95 (0.78)
Number of non-hydrogen atoms	7250
macromolecules	6509
ligands	55
solvent	686
Protein residues	846
RMS(bonds)	0.010
RMS(angles)	1.35
Ramachandran favored (%)	98.57
Ramachandran allowed (%)	1.31
Ramachandran outliers (%)	0.12
Average B-factor	43.48
macromolecules	43.21
ligands	47.43
solvent	45.65

Supplementary Table 2. Crystal structure statistics for the PUF60 UHM domain in complex with 7,8-dimethoxyperphenazine.

Supplementary Figure 1

a

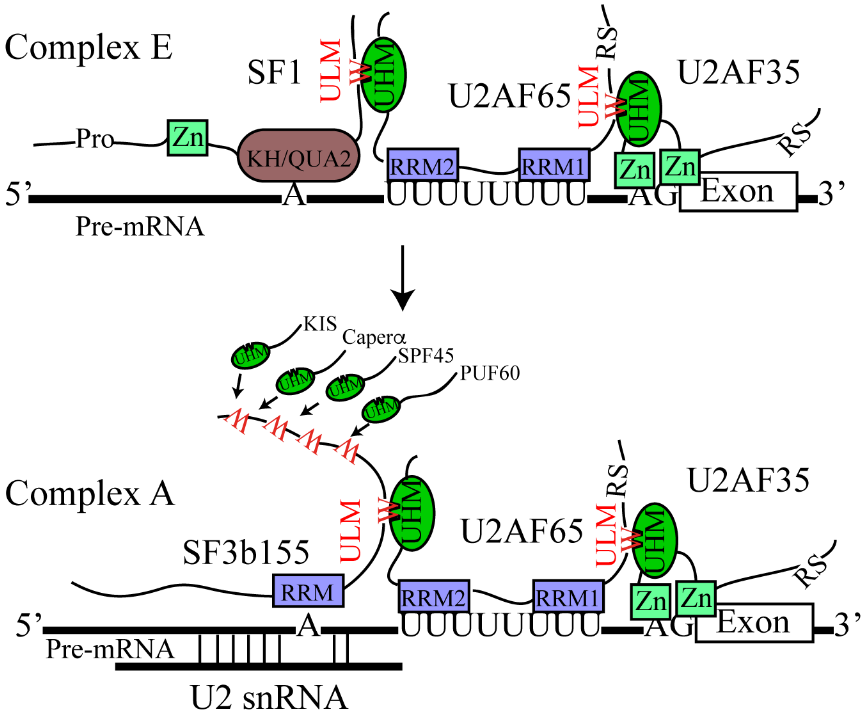

b

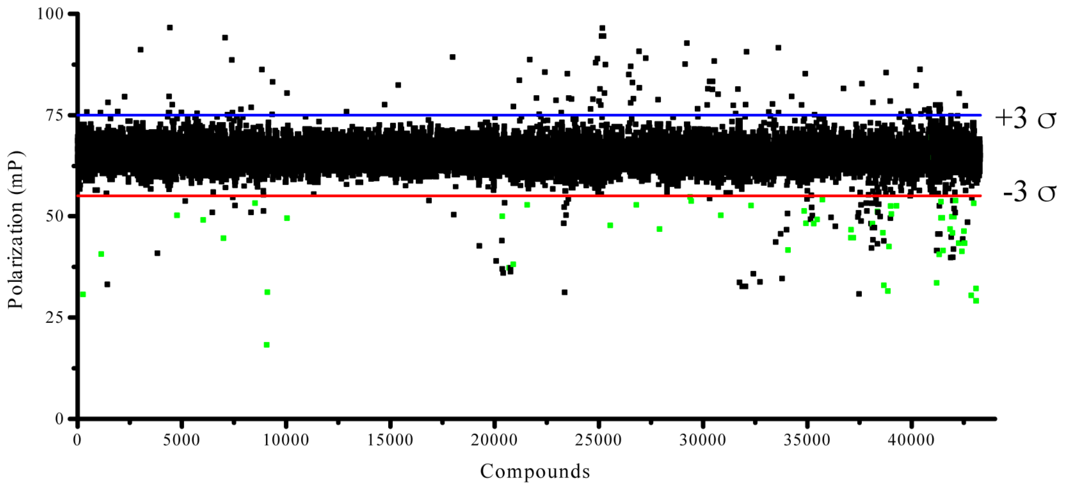

Supplementary Figure 1. a) Known UHM-ULM interactions involved in spliceosomal E and A complex formation are shown. The UHM domains and ULM peptide motifs are shown in green and red, respectively. b) Results of 2nd (duplicate) HTS campaign. Compounds that show a decrease in the polarization of light by 3-fold standard deviation from the mean plate value and are reproducible in duplicate HTS campaigns are shown in green.

Supplementary Figure 2

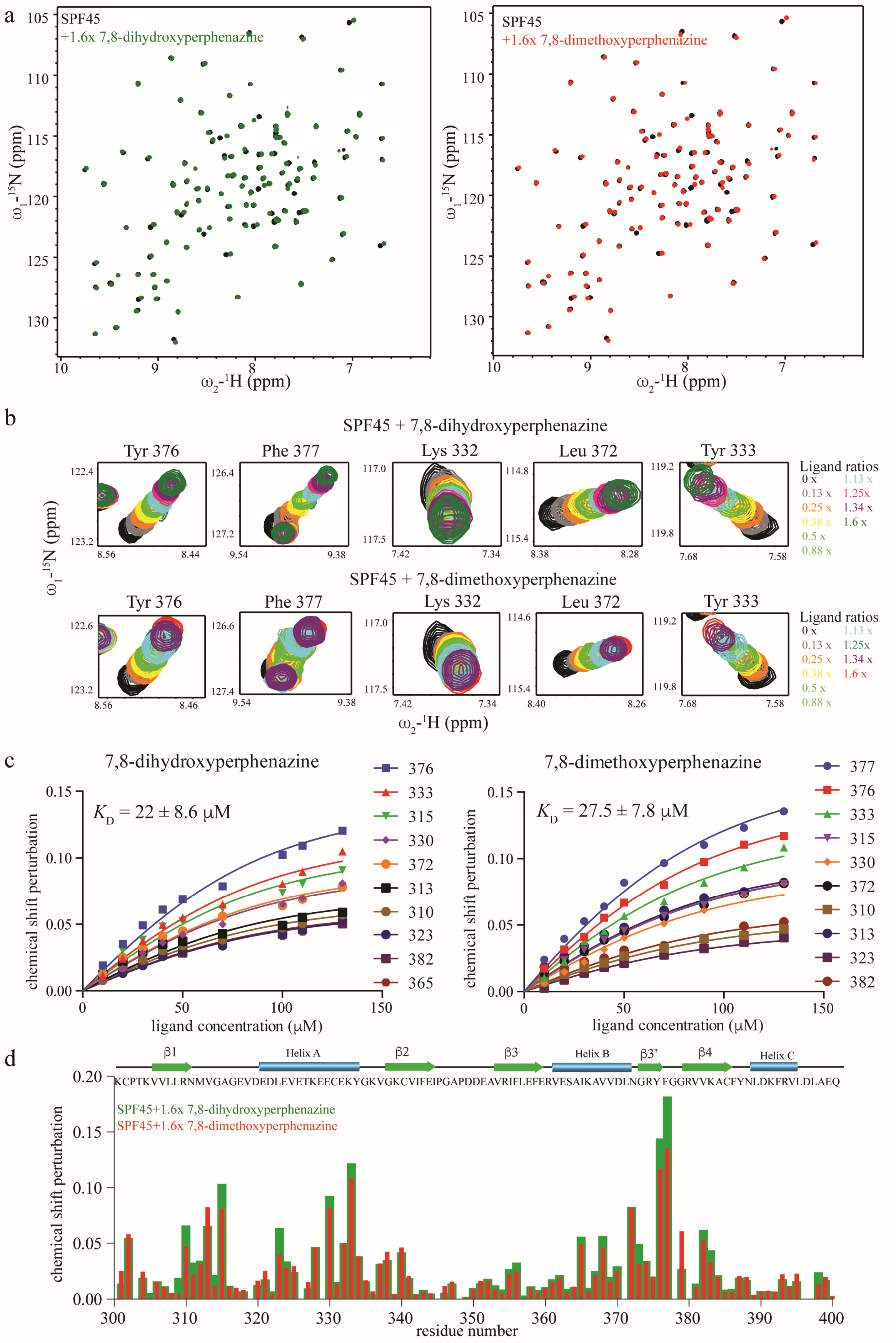

Supplementary Figure 2. a) ^{1}H-^{15}N HSQC spectra of the SPF45 UHM domain free and in the presence of saturating concentration (1.6-fold molar excess) of 7,8- dihydroxyperphenazine (left) and 7,8-dimethoxyperphenazine (right). Residues that form the binding site are annotated. b) NMR titration focusing on amide signals for residues in the binding site for 7,8-dihydroxyperphenazine (top) and 7,8-dimethoxyperphenazine (bottom). c) Binding affinity curves for 7,8- dihydroxyperphenazine and 7,8-dimethoxyperphenazine obtained from NMR titration experiments. d) CSPs vs. residue number for SPF45 UHM domain bound to equimolar 7,8-dimethoxyperphenazine.

Supplementary Figure 3

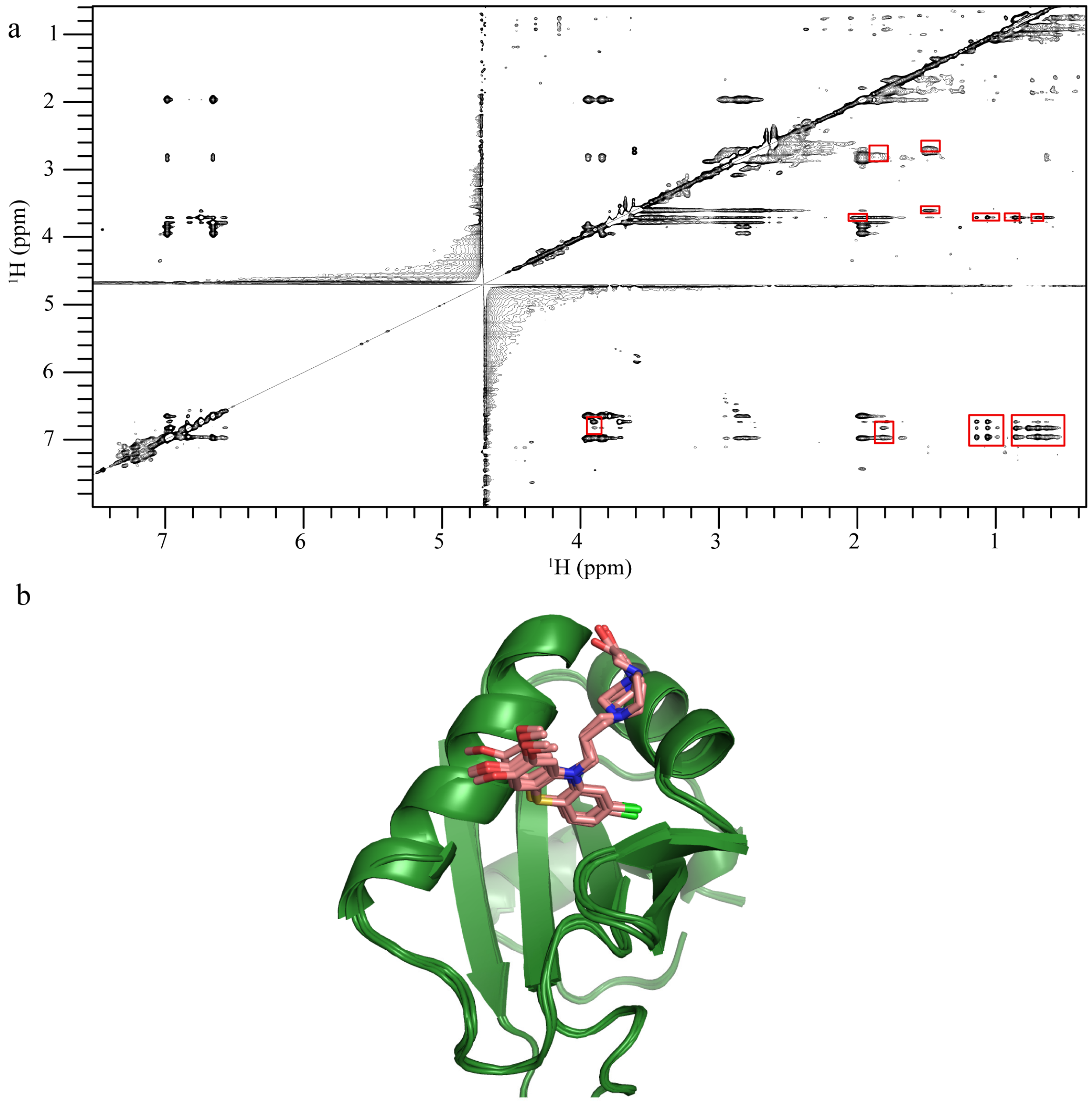

Supplementary Figure 3. a) 2D ω_1-filtered NOESY experiment for 650 µM sample of ^{15}N,^{13}C-labeled SPF45 UHM domain bound to 1.3 mM 7,8-dimethoxyperphenazine, recorded at a proton Larmor frequency of 600 MHz, T=298 K, with a mixing time of 150 ms. Intermolecular NOEs are highlighted by red boxes. b) Superposition of the four best models of the SPF45 UHM domains with lowest energy score from the HADDOCK calculation. Note, that due to technical limitations of semi-flexible docking, the expansion of the tryptophan-binding pocket of SPF45 UHM domain to accommodate the phenothiazine moiety is limited and thus the phenothiazine moiety remains planar in the docked structure.

Supplementary Figure 4

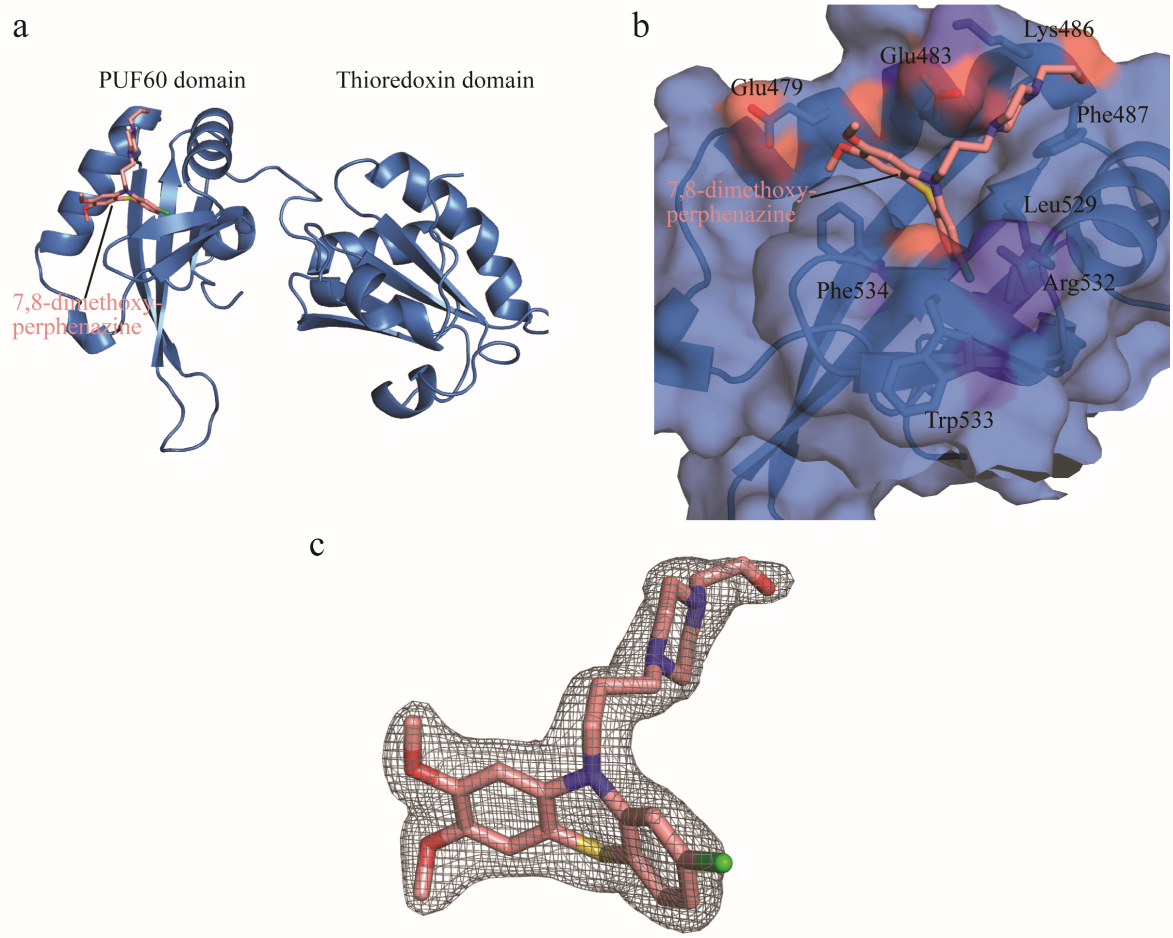

Supplementary Figure 4. a) Crystal structure of thioredoxin tagged PUF60 UHM domain in complex with 7,8-dimethoxyperphenazine (shown in salmon color). b) Surface representation of the 7,8-dimethoxyperphenazine binding site on the PUF60 UHM along with the residues that interact with the inhibitor. c) $2F_o$-F_c map of 7,8-dimethoxyperphenazine contoured at 1σ.

Supplementary Figure 5

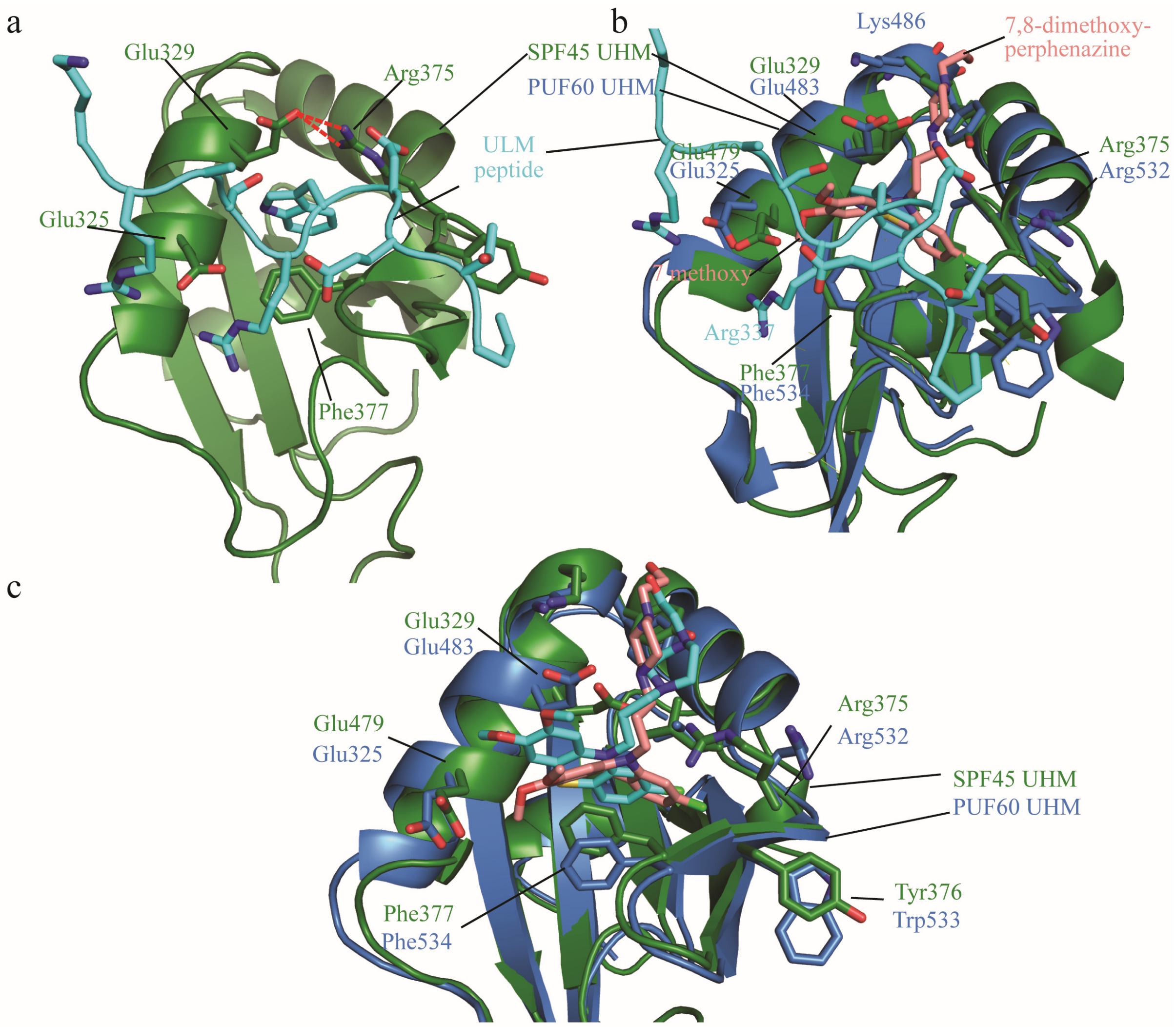

Supplementary Figure 5. a) The previously reported crystal structure of the SPF45 UHM domain (green) in complex with a SF3b1-ULM peptide (cyan color) (PDB ID. 2PEH), with side chains of residues involved in ULM binding shown. Note that Glu329 forms hydrogen bonds with Arg375, thus forming a part of the tryptophan binding site of ULM. b) Superposition of the SPF45 UHM/SF3b-ULM structure with the PUF60 UHM domain (blue) bound to 7,8-dimethoxyperphenazine (salmon). PUF60 Glu483 and Arg532 (corresponding to position Glu329 and Arg375 in SPF45 UHM) are unable to form hydrogen bonds as the piperazine moiety extends in the direction opposite to the tryptophan binding site. In addition, the 7-methoxy group of 7,8-dimethoxyperphenazine points in the direction of the pocket occupied by Arg337 in the ULM peptide. c) Superposition of the crystal structure of PUF60 UHM domain (blue) bound to 7,8-dimethoxyperphenazine (salmon) with the HADDOCK model of SPF45 UHM domain (green) bound to 7,8-dimethoxyperphenazine (cyan). The ligands in the two structures occupy the same site and show a similar binding mode. However, as the binding pocked is not able to expand during the semi-flexible HADDOCK docking, the 7,8-dimethoxyperphenazine in the SPF45 UHM domain does not enter completely into the tryptophan-binding pocket.

Supplementary Figure 6

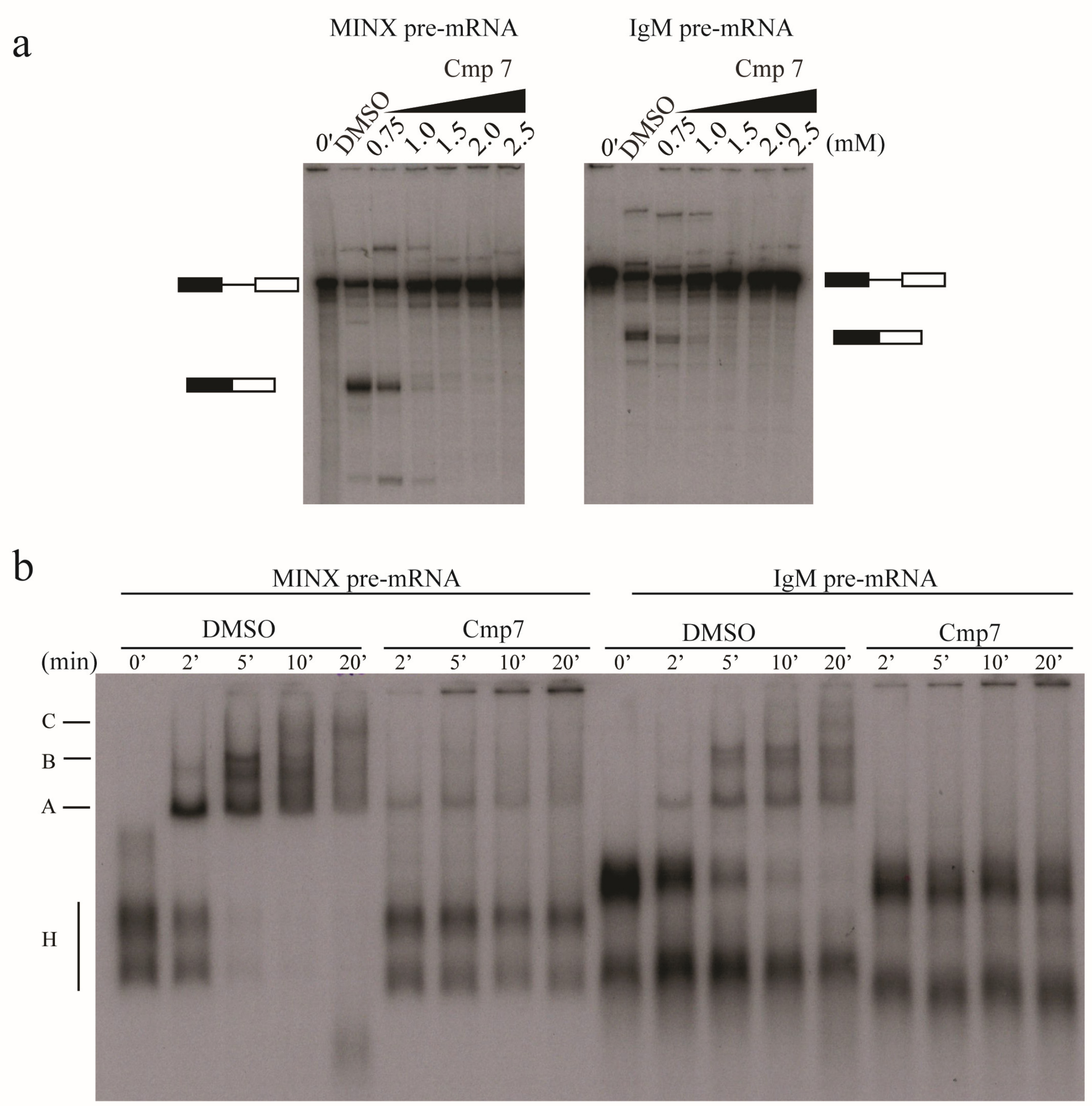

Supplementary Figure 6. *In vitro* splicing assay of MINX and IgM pre-mRNA with 7,8-dimethoxyperphenazine (Cmp7). a) 7,8-dimethoxyperphenazine (Cmp7) abolishes splicing of MINX pre-mRNA at 2.5 mM and of IgM pre-mRNA at 2 mM. b) Separation of spliceosomal complexes on an agarose gel. A complex formation of IgM is essentially completely abolished at 2 mM of Cmp7 while with MINX very low levels of the A complex were still observed at 2 mM. The effect of Cmp7 on MINX and IgM pre-mRNA splicing and splicing complex formation in vitro was tested in 2 or 3 independent experiments with similar results. The Uncropped gel images are included in the Source Data file.

Supplementary Methods

Synthesis of the cyclic peptide tagged with fluorescein for HTS

The tracer peptide [sc,sc(KSRWDE)]-K-C-fluorescein was used for fluorescence polarization assays (**Figure 1**). The tracer is based on our recently reported cyclic peptide [sc,sc (KSRWDE)]-K, which binds to the SFP45 UHM domain with high affinity [1].

Synthesis of the precursor [sc,sc(Boc-KS(tBu)R(Pbf)W(Boc)- D(OtBu)E)]-OH

The orthogonally protected, linear peptide Boc-Lys(Fmoc)-Ser(tBu)-Arg(Pbf)-Trp(Boc)-Asp(OtBu)-Glu(OAllyl)-OH was synthesized on a solid support (trityl chloride resin, Intavis, Germany) using a standard Fmoc procedure. Couplings were performed using 2 equiv of amino acid, 2 equiv of HATU, 2 equiv of HOAt, and 5 equiv of DIPEA. After final Fmoc and Allyl deprotection on solid support, the side chains of the peptide's lysine and glutamic acid were cyclized on the resin by incubating the orthogonally deprotected peptide for 1 h with a solution of 2 equiv of HATU, 2 equiv of HOAt, and 5 equiv of DIPEA in DMF. After completion of the reaction (monitoring by LC-MS), the peptide was cleaved from the resin using a 20% HFIP solution in DCM. After evaporation of the solvent, the product was dissolved in H_2O/ACN and lyophilized overnight. The peptide [*sc,sc*(Boc-KS(tBu)R(Pbf)W- (Boc)D(OtBu)E)]-OH was obtained in good purity (>90%) as a white powder.

Synthesis of peptide precursor [sc,sc(KSRWDE)]-K-C-OH

Fmoc-Cys(Trt) was loaded onto trityl chloride resin (Intavis, Germany). After Fmoc deprotection, Fmoc-Lys(Boc)-OH was coupled using 2 eq. HATU, 2 eq. HOAt and 5 eq. DIPEA for 2 h in DMF. After Fmoc-Deprotection, the resin was incubated with a solution of the compound [sc,sc(Boc-KS(tBu)R-(Pbf)W(Boc)D(OtBu)E)]-OH (1.5 equiv), HATU (1.5 equiv), HOAt (1.5 equiv), and DIPEA (3 equiv) in DMF until all resin-bound starting material was consumed (at least 3 h). The reaction was monitored using LC-MS. After completion of the reaction, the coupled peptide was cleaved from the resin (20% HFIP/DCM) and subsequently treated with a solution of 85% TFA, 10% DCM, 2.5% TIPS, and 2.5% H_2O to deprotect the acid-labile side chain protecting groups. Afterward, the peptide was purified by semipreparative HPLC and lyophilized, yielding a colorless powder. The peptide was obtained in good purity (>90%).

ESI-MS (found): $[M+H]^+$ = 1033.5.

HPLC: t_R = 4.4 min (RP C18, Gradient 5−95%, 8 min).

Conjugation to fluorescein

N-(5-Fluoresceinyl)maleimide is dissolved at a concentration of 5 mg/mL in DMF. This solution is slowly added to a solution of peptide precursor in PBS buffer at pH 7.0 (concentration 5 mg/mL). The solution is stirred for 1 h at room temperature and the labeled peptide purified by semipreparative HPLC (stationary phase YMC C18) and lyophilized.

Synthesis of the phenothiazine inhibitors

General Conditions

Unless otherwise noted, all reagents were obtained from commercial sources and used without further purification. Technical grade solvents used for aqueous workup were distilled prior to use. Dry tetrahydrofuran and methanol were purchased from Acros. Analytical thin layer chromatography (TLC) was performed on silica (silica gel 60 F 254) coated plates. Compounds were detected by ultraviolet (UV) irradiation at 254 or 366 nm. High-resolution mass spectrometry (HRMS) measurements were performed on a Thermo Finnigan LTQ FT apparatus using an electrospray ionization (ESI) detector. NMR spectra were recorded at 303 K on a Bruker Avance III HD 400 (400 MHz) spectrometer. Chemical shifts are reported in parts per million (ppm) relative to residual *d*5-DMSO (δH = 2.50 ppm) and *d*6-DMSO (δC = 39.52 ppm). Splitting patterns are designated as s (singlet), d (doublet), t (triplet), td (triplet of doublets), ddd (doublet of doublets of doublets), m (multiplet) or bs (broad signal). The coupling constants (J) are reported in Hertz (Hz).

General procedure 1: *O*-alkylation of 2-chloro-10*H*-phenothiazin-7-ol

The flask containing 2-chloro-8-methoxy-10*H*-phenothiazin-7-ol (100 mg, 0.36 mmol), KOH (40 mg, 0.72 mmol, 2 equiv.) and $Na_2S_2O_4$ (250 mg, 1.44 mmol, 4 equiv., 85% purity) was evacuated and back-filled with Argon. Acetone (4 mL) was added and the mixture was stirred for 30 min at rt. The alkylating reagent (2 equiv.) was added in one portion, reaction flask was covered with aluminium foil to avoid the access of the light and refluxed for 15 hours. Purification by column chromatography using the gradient from Hexane to Hexane:EtOAc 1:1 (SiO_2, solid loading) gave the title compound.

General procedure 2: *N*-alkylation of 2-chloro-8-methoxy-10*H*-phenothiazines

The flask containing derivative of phenothiazine (0.29 mmol), 2-(4-(3-chloropropyl)piperazin-1-yl)ethan-1-ol (0.29 mmol, 1 equiv.), NaH (0.58 mmol, 2 equiv., 60% dispersion in mineral oil) and KI (0.29 mmol, 1 equiv.) was evacuated and back-filled with Argon. DMF (5 mL) was added, the reaction flask was covered with aluminum foil to avoid the access of the light and heated to 80°C for 15 hours. The reaction was quenched with H_2O (25 mL) and diluted with EtOAc (25 mL). The layers were separated and the aqueous layer was extracted twice with EtOAc (25 mL). The combined organic layers were dried ($MgSO_4$) and concentrated *in vacuo*. Purification by column chromatography using the gradient from $CHCl_3$ to $CHCl_3$:MeOH 10:1 (SiO_2, dry load) gave the title compound.

Cmp1

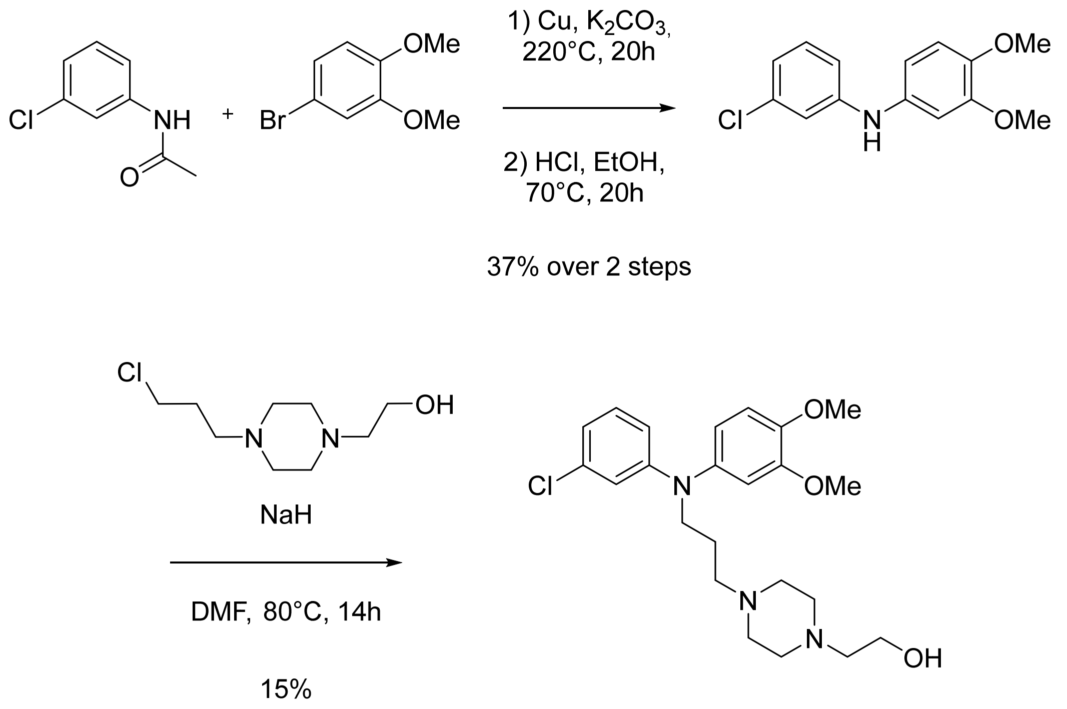

Scheme 1: Synthesis of the cmp1.

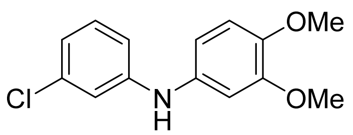

***N*-(3-chlorophenyl)-3,4-dimethoxyaniline**

A mixture of *N*-(3-chlorophenyl)acetamide (4.69 g; 0.028 mol), 4-bromo-1,2-dimethoxybenzene (3,31 mL; 0.023 mol), Cu (80 mg) and K_2CO_3 (2.23 g; 0.016 mol) was refluxed under condenser at 220°C. According to TLC was after 20 hours all starting material consumed. Obtained brown oil was dissolved in acetone and evaporated. The reaction mixture was dissolved in EtOH (25 mL), HCl was added (9 mL) and heated to 70°C for further 20 hours. The mixture was allowed to cool to rt, poured into water (50 ml), neutralized by addition of the solution of 2M NaOH and diluted with Et_2O (25 mL). The layers were separated and the aqueous layer was extracted twice with Et_2O (25 mL). The combined organic layers were dried ($MgSO_4$) and concentrated *in vacuo.* Purification by column chromatography using the gradient from hexane to hexane:EtOAc 4:1 (SiO_2, dry load) gave *N*-(3-chlorophenyl)-3,4-dimethoxyaniline (2.245 g; 37% over two steps) as a pale orange oil which spontaneously crystalized.

^{1}H NMR (360 MHz, DMSO-d_6) δ 8.11 (bs, 1H), 7.15 (t, J = 8.0 Hz, 1H), 6.92 – 6.83 (m, 3H), 6.73 – 6.64 (m, 3H), 3.73 (s, 3H), 3.72 (s, 3H).

^{13}C NMR (91 MHz, DMSO) δ 149.42, 146.96, 144.24, 135.37, 133.60, 130.64, 117.43, 113.62, 112.98, 112.95, 111.69, 105.63, 55.90, 55.42.

HRMS (+ESI) for $C_{14}H_{14}ClNO_2$: $[M + H]^+$ calculated, 264,0791; found, 264,0786.

m.p. 102°C

The synthesis of *N*-(3-chlorophenyl)-3,4-dimethoxyaniline was performed as reported previously [2]. Unfortunately, no spectral data are provided for the title compound. Only m.p. 101,5-102°C is given, which agrees with our data.

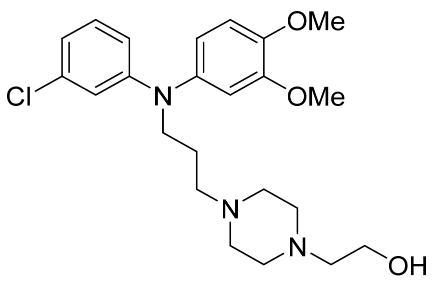

2-(4-(3-((3-chlorophenyl)(3,4-dimethoxyphenyl)amino)propyl)piperazin-1-yl)ethan-1-ol

The flask containing *N*-(3-chlorophenyl)-3,4-dimethoxyaniline (194 mg; 0.74 mmol), 2-(4-(3-chloropropyl)piperazin-1-yl)ethan-1-ol (153 mg; 0.74 mmol, 1 equiv.) and NaH (22 mg; 0.88 mmol, 1.2 equiv., 60% dispersion in mineral oil) was evacuated and back-filled with Argon. DMF (7 mL) was added and reaction mixture was heated to 80°C for 14 hours. The reaction was quenched with H_2O (25 mL) and diluted with EtOAc (25 mL). The layers were separated and the aqueous layer was extracted twice with EtOAc (25 mL). The combined organic layers were dried ($MgSO_4$) and concentrated *in vacuo*. Purification by column chromatography using the gradient from $CHCl_3$ to $CHCl_3$:MeOH 10:1 (SiO_2, dry load) gave the title compound (48 mg, 15%) as an orange oil.

^{1}H NMR (500 MHz, Chloroform-*d*) δ 7.03 (t, *J* = 8.1 Hz, 1H), 6.87 (d, *J* = 8.5 Hz, 1H), 6.76 – 6.73 (m, 1H), 6.72 (dd, *J* = 8.5, 2.3 Hz, 1H), 6.69 – 6.66 (m, 2H), 6.56 (dd, *J* = 8.4, 1.9 Hz, 1H), 3.90 (s, 3H), 3.82 (s, 3H), 3.70 – 3.62 (m, 4H), 2.70 – 2.38 (m, 12H), 1.84 – 1.76 (m, 2H).

^{13}C NMR (91 MHz, Chloroform) δ 150.33, 149.94, 146.97, 139.62, 134.86, 129.78, 119.12, 117.33, 114.44, 112.92, 112.02, 110.85, 59.41, 57.52, 56.09, 56.02, 55.33, 52.85, 52.82, 50.22, 24.69.

HRMS (+ESI) for $C_{23}H_{32}ClN_3O_3$: $[M + H]^+$ calculated, 434,2210; found, 434,2219.

Cmp2

The compound is literature known and was prepared according to a reported procedure [3].

Cmp3

This compound Perphenazine was purchased from Sigma Aldrich (product number P6402)

Cmp4

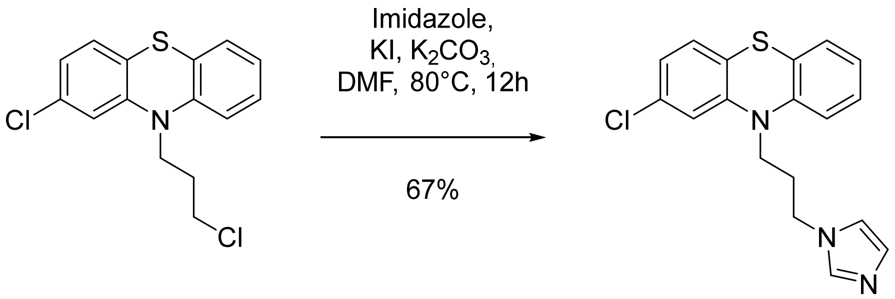

Scheme 2: Synthesis of the cmp4.

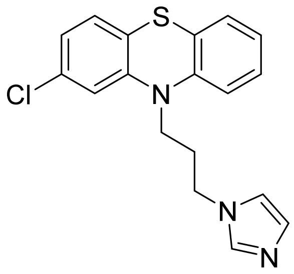

10-(3-(1*H*-imidazol-1-yl)propyl)-2-chloro-10*H*-phenothiazine

The flask containing 2-chloro-10-(3-chloropropyl)-10*H*-phenothiazine (160 mg; 0.52 mmol), benzimidazole (183 mg; 1.55 mmol; 3 equiv.), K_2CO_3 (86 mg; 0.62 mmol; 1.2 equiv.) and KI (86 mg; 0.52 mmol, 1 equiv.) was evacuated and back-filled with Argon. DMF (10 mL) was added and the reaction mixture was heated to 80°C for 12 hours. The reaction was quenched with H_2O (25 mL) and diluted with EtOAc (25 mL). The layers were separated and the aqueous layer was extracted twice with EtOAc (25 mL). The combined organic layers were dried ($MgSO_4$) and concentrated *in vacuo*. Purification by column chromatography using the gradient from CH_2Cl_2 to CH_2Cl_2:MeOH 20:1 (SiO_2, dry load) gave the title compound (165 mg, 82%) as a pink oil.

^{1}H NMR (360 MHz, Chloroform-*d*) δ 7.31 (s, 1H), 7.24 – 7.08 (m, 3H), 7.04 – 6.92 (m, 3H), 6.85 – 6.72 (m, 3H), 4.05 (t, J = 6.5 Hz, 2H), 3.79 (t, J = 6.1 Hz, 2H), 2.22 (p, J = 6.3 Hz, 2H).

^{13}C NMR (91 MHz, $CDCl_3$) δ 146.31, 144.42, 137.55, 133.63, 129.70, 128.45, 128.03, 127.83, 126.06, 124.87, 123.67, 123.12, 119.11, 116.42, 116.33, 43.43, 43.28, 28.09.

HRMS (+ESI) for $C_{18}H_{16}ClN_3S$: $[M + H]^+$ calculated, 342,0832; found, 342,0825.

Cmp5

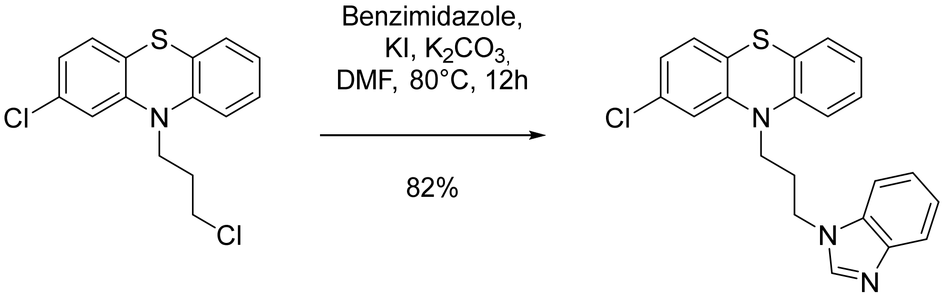

Scheme 3: Synthesis of the cmp5.

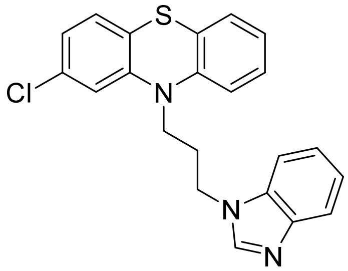

10-(3-(1*H*-benzo[*d*]imidazol-1-yl)propyl)-2-chloro-10*H*-phenothiazine

The flask containing 2-chloro-10-(3-chloropropyl)-10*H*-phenothiazine (128 mg; 0.41 mmol), imidazole (84 mg; 1.24 mmol; 3 equiv.), K_2CO_3 (68 mg; 0.49 mmol; 1.2 equiv.) and KI (68 mg; 0.41 mmol, 1 equiv.) was evacuated and back-filled with Argon. DMF (10 mL) was added and the reaction mixture was heated to 80°C for 12 hours. The reaction was quenched with H_2O (25 mL) and diluted with EtOAc (25 mL). The layers were separated and the aqueous layer was extracted twice with EtOAc (25 mL). The combined organic layers were dried ($MgSO_4$) and concentrated *in vacuo*. Purification by column chromatography using the gradient from $CHCl_3$ to $CHCl_3$:MeOH 50:1 (SiO_2, dry load) gave the title compound (95 mg, 67%) as an orange oil.

^{1}H NMR (360 MHz, Chloroform-*d*) δ 7.76 (d, J = 7.9 Hz, 1H), 7.67 (s, 1H), 7.25 – 7.11 (m, 6H), 7.01 (td, J = 7.5, 0.9 Hz, 1H), 6.97 (dd, J = 8.2, 2.0 Hz, 1H), 6.78 (dd, J = 5.0, 3.0 Hz, 2H), 4.30 (t, J = 6.5 Hz, 2H), 3.83 – 3.77 (m, 2H), 2.40 – 2.28 (m, 2H).

^{13}C NMR (91 MHz, $CDCl_3$) δ 146.34, 144.37, 144.04, 143.49, 133.69, 133.61, 128.47, 128.06, 127.88, 126.22, 125.00, 123.75, 123.19, 122.96, 122.21, 120.57, 116.50, 116.43, 109.59, 43.36, 41.46, 26.45.

HRMS (+ESI) for $C_{22}H_{18}ClN_3S$: $[M + H]^+$ calculated, 392,0988; found, 392,0980.

Cmp6

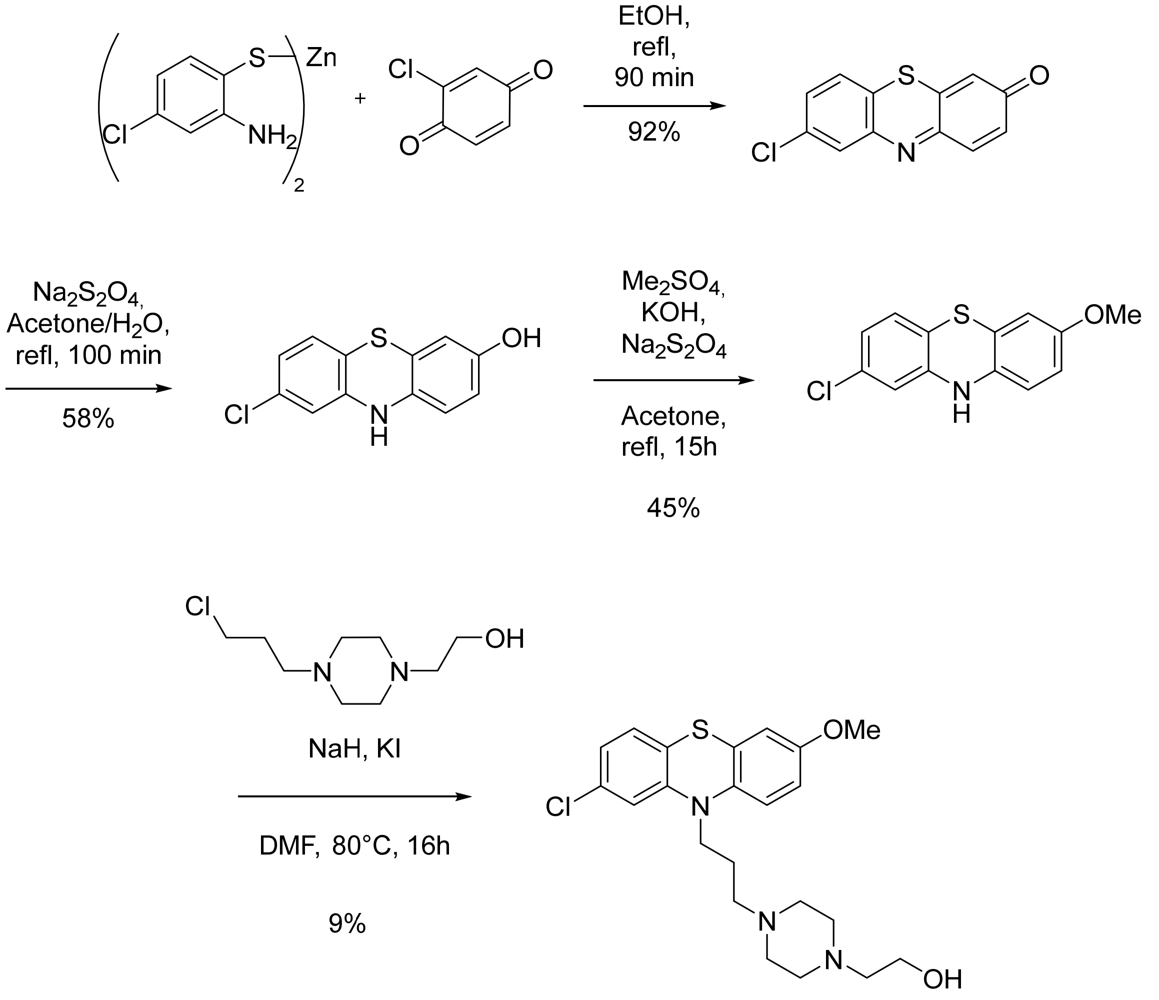

Scheme 4: Synthesis of the cmp6.

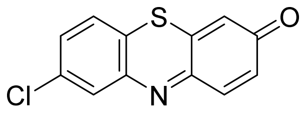

2-Chloro-7*H*-phenothiazin-7-one

A mixture of 2-chlorobenzoquinone (224 mg, 1.57 mmol), zinc salt of 2-amino-4-chlorobenzothiol (600 mg, 1.57 mmol) and 10 mL of EtOH was refluxed for 90 mins. The solid was filtrated, washed with EtOH (5 mL) and dried to give 2-chloro-7*H*-phenothiazin-7-one as a dark-red solid (420 mg, 92%).

^{1}**H NMR** (360 MHz, Chloroform-*d*) δ 7.92 (d, *J* = 2.2 Hz, 1H), 7.61 (d, *J* = 9.9 Hz, 1H), 7.46 (dd, *J* = 8.5, 2.2 Hz, 1H), 7.40 (d, *J* = 8.5 Hz, 1H), 6.97 (dd, *J* = 9.9, 2.2 Hz, 1H), 6.77 (d, *J* = 2.2 Hz, 1H).

^{13}**C NMR** (91 MHz, $CDCl_3$) δ 182.38, 147.48, 140.00, 139.72, 135.59, 134.62, 133.40, 133.13, 130.97, 125.86, 121.87, 120.47.

HRMS (+ESI) for $C_{12}H_6ClNOS$: $[M + H]^+$ calculated, 247.9937; found, 247.9921.

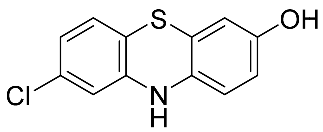

2-Chloro-10*H*-phenothiazin-7-ol

A mixture of $Na_2S_2O_4$ (1.05 g, 5.1 mmol, 3.1 equiv., 85% purity) and 2-chloro-7*H*-phenothiazin-7-one (408 mg, 1.65 mmol) was dissolved in acetone (15 mL) and H_2O (1.5 mL) and refluxed for 100 min. The mixture was allowed to cool to rt, poured into a solution of $Na_2S_2O_4$ (4 g) in H_2O (100 mL) and diluted with Et_2O (25 mL). The layers were separated and the aqueous layer was extracted twice with Et_2O (25 mL). The combined organic layers were dried ($MgSO_4$) and concentrated *in vacuo* to give 2-chloro-10*H*-phenothiazin-7-ol (237 mg, 58%) as a purple solid.

^{1}H NMR (360 MHz, DMSO-d_6) δ 9.07 (s, 1H), 8.44 (s, 1H), 6.89 (d, *J* = 8.2 Hz, 1H), 6.72 (dd, *J* = 8.2, 2.2 Hz, 1H), 6.65 (d, *J* = 2.1 Hz, 1H), 6.52 (d, *J* = 8.5 Hz, 1H), 6.44 (dd, *J* = 8.5, 2.6 Hz, 1H), 6.37 (d, *J* = 2.5 Hz, 1H).
^{13}C NMR (91 MHz, DMSO) δ 153.32, 144.86, 133.43, 132.16, 127.80, 120.81, 117.31, 115.88, 115.40, 114.74, 113.77, 113.30.
HRMS (+ESI) for $C_{12}H_8ClNOS$: $[M + H]^+$ calculated, 250.0093; found, 250.0085.

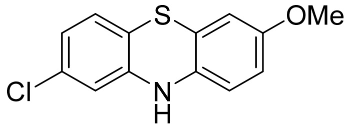

2-Chloro-7-methoxy-10*H*-phenothiazine

2-Chloro-7-methoxy-10*H*-phenothiazine was prepared from 2-chloro-10*H*-phenothiazin-7-ol (95 mg, 0.38 mmol) using Me_2SO_4 according to *General Procedure 1* to give the title compound (45 mg, 45%) as a dark red solid.

^{1}H NMR (500 MHz, DMSO-d_6) δ 8.57 (s, 1H), 6.93 (d, *J* = 8.2 Hz, 1H), 6.76 (dd, *J* = 8.2, 2.2 Hz, 1H), 6.67 (d, *J* = 2.2 Hz, 1H), 6.64 – 6.57 (m, 3H), 3.66 (s, 3H).
^{13}C NMR (126 MHz, DMSO) δ 155.41, 144.60, 134.88, 132.26, 127.90, 121.14, 117.64, 115.77, 115.34, 113.89, 113.73, 112.15, 55.86.
HRMS (+ESI) for $C_{13}H_{10}ClNOS$: $[M + H]^+$ calculated, 264.0250; found, 264.0241.

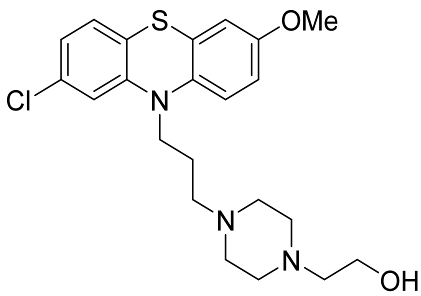

2-Chloro-7-methoxy-10-(3-(1-(2-hydroxyethyl)-4-piperazinyl)-propyl)phenothiazine

2-Chloro-7-methoxy-10-(3-(1-(2-hydroxyethyl)-4-piperazinyl)-propyl)phenothiazine was prepared from 2-chloro-7-methoxy-10*H*-phenothiazine (65 mg, 0.25 mmol) according to *General Procedure 2* to give the title compound (10 mg, 9%) as a brown oil.

^{1}H NMR (360 MHz, Chloroform-*d*) δ 7.03 (d, *J* = 8.1 Hz, 1H), 6.88 (dd, *J* = 8.1, 2.0 Hz, 1H), 6.84 (d, *J* = 2.0 Hz, 1H), 6.83 – 6.79 (m, 1H), 6.75 – 6.69 (m, 2H), 3.88 (t, *J* = 6.7 Hz, 2H), 3.77 (s, 3H), 3.70 – 3.64 (m, 2H), 2.74 – 2.42 (m, 12H), 2.01 – 1.90 (m, 2H).
^{13}C NMR (91 MHz, $CDCl_3$) δ 155.64, 147.03, 137.67, 133.25, 127.85, 126.29, 123.18, 121.87, 116.43, 115.63, 112.95, 112.85, 59.44, 57.42, 55.70, 55.20, 52.72, 52.60, 45.26, 24.06.
HRMS (+ESI) for $C_{22}H_{28}ClN_3O_2S$: $[M + H]^+$ calculated, 434.1664; found, 434.1665.

Cmp7

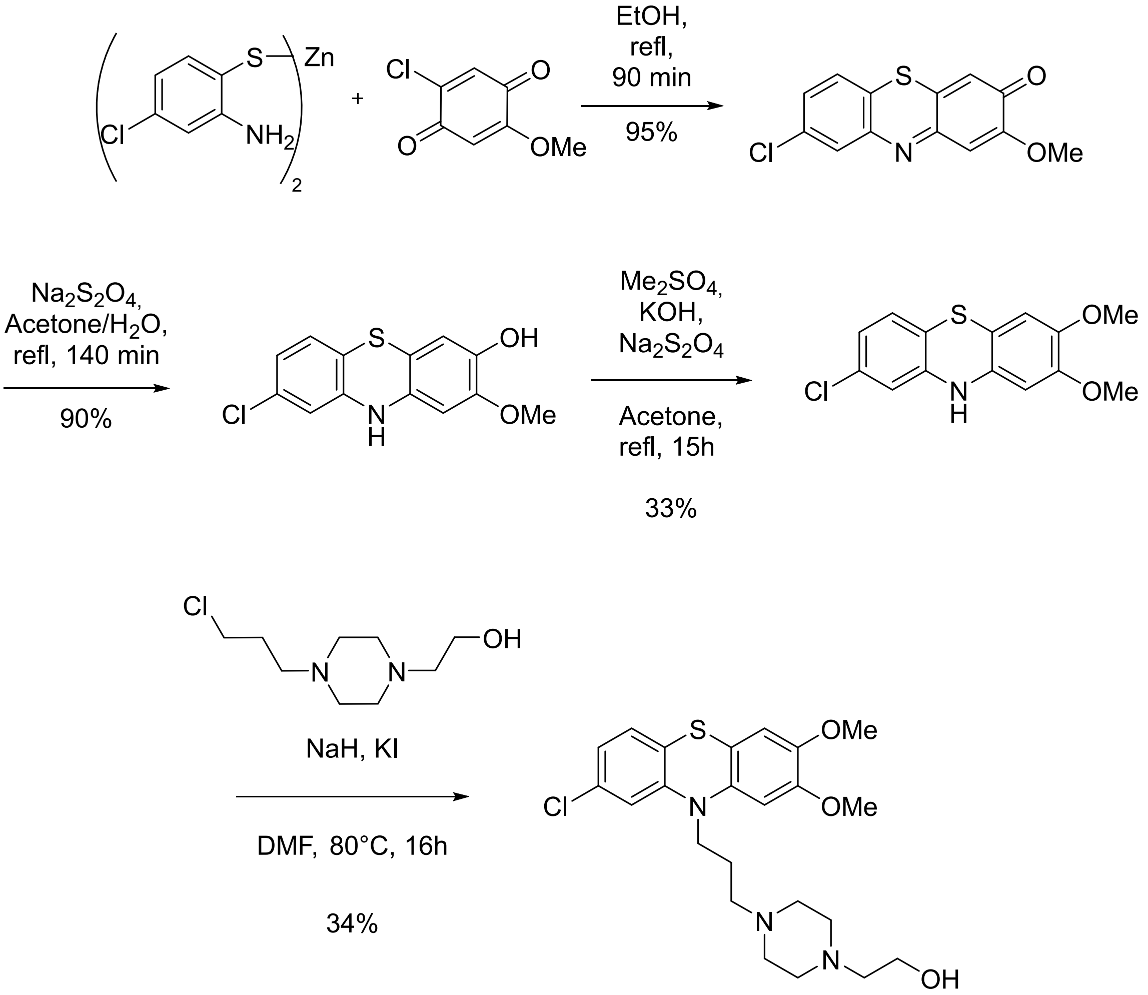

Scheme 5: Synthesis of the cmp7.

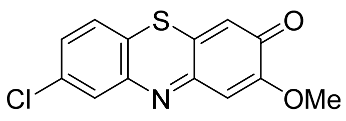

2-Chloro-8-methoxy-7*H*-phenothiazin-7-one

A mixture of 2-chloro-5-methoxybenzoquinone (1.03 g, 5.97 mmol), zinc salt of 2-amino-4-chlorobenzothiol (1.6 g, 4.18 mmol, 0.7 equiv) and 35 mL of EtOH was refluxed for 90 min.

The red-orange solid was filtrated, washed with EtOH (10 mL) and dried to give 2-chloro-8-methoxy-7*H*-phenothiazin-7-one as a red-orange solid (1.58 g, 95%).

^{1}H NMR (360 MHz, Chloroform-*d*) δ 7.95 – 7.83 (m, 1H), 7.46 – 7.38 (m, 2H), 6.86 (s, 1H), 6.83 (s, 1H), 3.98 (s, 3H).
^{13}C NMR (91 MHz, $CDCl_3$) δ 176.38, 157.90, 148.02, 140.08, 134.03, 133.36, 132.15, 129.66, 125.76, 120.35, 119.13, 110.61, 77.35, 77.20, 77.00, 76.64, 56.26.
HRMS (+ESI) for $C_{13}H_8ClNO_2S$: $[M + H]^+$ calculated, 278.0043; found, 278.0034.

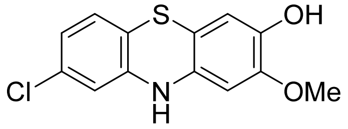

2-Chloro-8-methoxy-10*H*-phenothiazin-7-ol

A mixture of $Na_2S_2O_4$ (1.4 g, 6.85 mmol, 2.5 equiv., 85% purity) and 2-chloro-8-methoxy-7*H*-phenothiazin-7-one (760 mg, 2.74 mmol) was dissolved in acetone (50 mL) and H_2O (4 mL) and refluxed for 140 min. The mixture was allowed to cool to rt and poured into a solution of $Na_2S_2O_4$ (4 g) in H_2O (100 mL). The precipitate was filtrated, washed with H_2O (20 mL) and dried to give 2-chloro-8-methoxy-10*H*-phenothiazin-7-ol (689 mg, 90%) as a white-grey solid.

^{1}H NMR (360 MHz, DMSO-d_6) δ 8.66 (s, 1H), 8.44 (s, 1H), 6.89 (d, *J* = 8.2 Hz, 1H), 6.73 (dd, *J* = 8.2, 2.2 Hz, 1H), 6.65 (d, *J* = 2.2 Hz, 1H), 6.38 (s, 1H), 6.35 (s, 1H), 3.70 (s, 3H).
^{13}C NMR (91 MHz, DMSO) δ 147.77, 144.90, 142.57, 133.94, 132.00, 127.81, 121.03, 116.25, 113.85, 113.74, 106.18, 101.10, 56.13.
HRMS (+ESI) for $C_{13}H_{10}ClNO_2S$: $[M + H]^+$ calculated, 280.0199; found, 280.0146.

Synthesis of 2-chloro-8-methoxy-7*H*-phenothiazin-7-one and 2-chloro-8-methoxy-10*H*-phenothiazin-7-ol is published in the paper describing the synthesis of chlorpromazine metabolites [2].

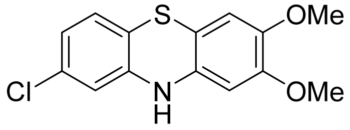

2-Chloro-7,8-dimethoxy-10*H*-phenothiazine

2-Chloro-7,8-dimethoxy-10*H*-phenothiazine was prepared from 2-chloro-8-methoxy-10*H*-phenothiazin-7-ol (200 mg, 0.71 mmol) using Me_2SO_4 according to *General Procedure 1* to give the title compound (70 mg, 33%) as a white solid.

^{1}H NMR (360 MHz, DMSO-d_6) δ 8.54 (s, 1H), 6.92 (d, *J* = 8.2 Hz, 1H), 6.76 (dd, *J* = 8.2, 2.2 Hz, 1H), 6.66 (d, *J* = 2.2 Hz, 1H), 6.58 (s, 1H), 6.38 (s, 1H), 3.70 (s, 3H), 3.65 (s, 3H).
^{13}C NMR (91 MHz, DMSO) δ 149.09, 145.06, 144.69, 135.46, 132.08, 127.85, 121.35, 116.29, 113.97, 111.50, 105.79, 100.96, 56.72, 56.02.
HRMS (+ESI) for $C_{14}H_{12}ClNO_2S$: $[M + H]^+$ calculated, 294.0350; found, 294.0349.

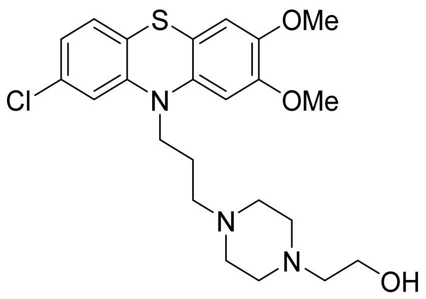

2-Chloro-7,8-dimethoxy-10-(3-(1-(2-hydroxyethyl)-4-piperazinyl)-propyl)phenothiazine

2-Chloro-7,8-dimethoxy-10-(3-(1-(2-hydroxyethyl)-4-piperazinyl)-propyl)phenothiazine was prepared from 2-chloro-7,8-dimethoxy-10*H*-phenothiazine (55 mg, 0.19 mmol) according to *General Procedure 2* to give the title compound (30 mg, 34%) as a yellow oil.

^{1}H NMR (400 MHz, Chloroform-*d*) δ 7.05 (d, *J* = 8.2 Hz, 1H), 6.91 (dd, *J* = 8.1, 2.0 Hz, 1H), 6.87 (d, *J* = 2.1 Hz, 1H), 6.70 (s, 1H), 6.53 (s, 1H), 3.97 – 3.91 (m, 2H), 3.89 (s, 3H), 3.85 (s, 3H), 3.77 – 3.71 (m, 2H), 2.87 – 2.56 (m, 12H), 2.05 – 1.97 (m, 2H).
^{13}C NMR (126 MHz, $CDCl_3$) δ 148.75, 147.06, 145.38, 138.00, 133.16, 127.96, 124.41, 122.27, 116.01, 115.40, 110.98, 101.85, 59.78, 57.16, 56.45, 56.38, 54.88, 52.55, 51.91, 45.22, 24.02.
HRMS (+ESI) for $C_{23}H_{30}ClN_3O_3S$: $[M + H]^+$ calculated, 464.1775; found, 464.1766.

Cmp8

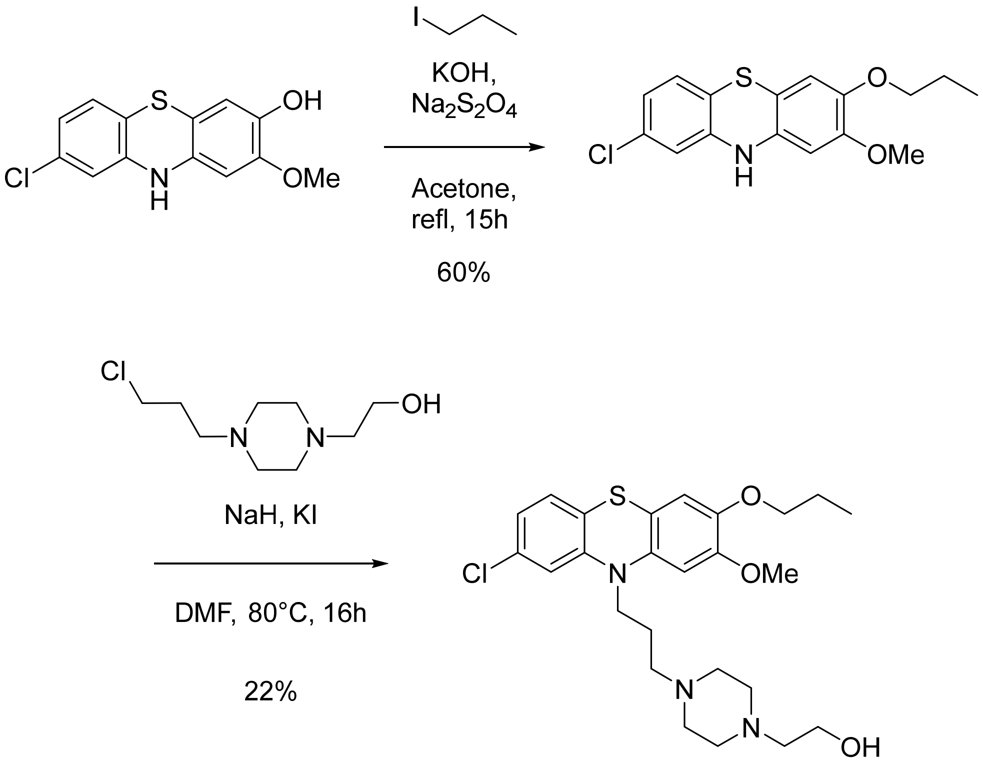

Scheme 6: Synthesis of the cmp8.

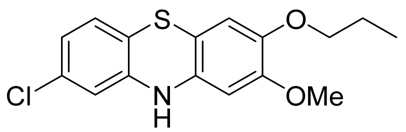

2-Chloro-8-methoxy-7-propoxy-10*H*-phenothiazine

2-Chloro-8-methoxy-7-propoxy-10*H*-phenothiazine was prepared from 2-chloro-8-methoxy-10*H*-phenothiazin-7-ol (200 mg, 0.71 mmol) using propyliodide according to *General Procedure 1* to give the title compound (139 mg, 60%) as a white solid.

^{1}H NMR (500 MHz, DMSO-d_6) δ 8.56 (s, 1H), 6.91 (d, *J* = 8.2 Hz, 1H), 6.76 (dd, *J* = 8.2, 2.2 Hz, 1H), 6.66 (d, *J* = 2.2 Hz, 1H), 6.57 (s, 1H), 6.37 (s, 1H), 3.78 (t, *J* = 6.6 Hz, 2H), 3.70 (s, 3H), 1.71 – 1.60 (m, 2H), 0.93 (t, *J* = 7.4 Hz, 3H).
^{13}C NMR (126 MHz, DMSO) δ 149.38, 144.69, 144.23, 135.52, 132.07, 127.89, 121.35, 116.30, 113.95, 112.79, 105.73, 100.93, 70.98, 56.00, 22.63, 10.90.
HRMS (+ESI) for $C_{16}H_{16}ClNO_2S$: [M + H]$^+$ calculated, 322.0663; found, 322.0663.

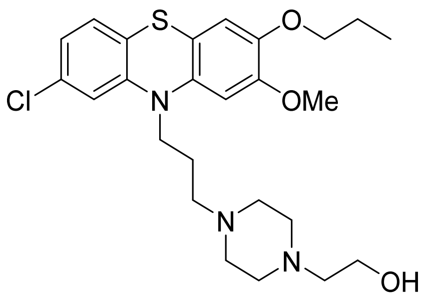

2-Chloro-8-methoxy-7-propoxy-10-(3-(1-(2-hydroxyethyl)-4-piperazinyl)-propyl)phenothiazine

2-Chloro-8-methoxy-7-propoxy-10-(3-(1-(2-hydroxyethyl)-4-piperazinyl)-propyl)phenothiazine was prepared from 2-chloro-8-methoxy-7-propoxy-10*H*-phenothiazine (121 mg, 0.38 mmol) according to *General Procedure 2* to give the title compound (40 mg, 22%) as a yellow oil.

^{1}H NMR (360 MHz, Chloroform-*d*) δ 7.01 (s, 1H), 6.86 (dd, *J* = 8.1, 2.0 Hz, 1H), 6.84 (d, *J* = 2.1 Hz, 1H), 6.67 (s, 1H), 6.50 (s, 1H), 3.92 – 3.87 (m, 4H), 3.84 (s, 3H), 3.63 – 3.58 (m, 2H), 2.60 – 2.32 (m, 12H), 1.98 – 1.90 (m, 2H), 1.82 (q, *J* = 7.1 Hz, 2H), 1.01 (t, *J* = 7.4 Hz, 3H).
^{13}C NMR (91 MHz, $CDCl_3$) δ 149.34, 147.09, 144.96, 138.35, 133.08, 127.80, 124.18, 122.02, 115.87, 115.50, 113.22, 102.70, 71.37, 59.22, 57.67, 56.75, 55.37, 53.22, 52.84, 45.51, 24.57, 22.54, 10.39.
HRMS (+ESI) for $C_{25}H_{34}ClN_3O_3S$: [M + H]$^+$ calculated, 492.2082; found, 492.2081.

Cmp9

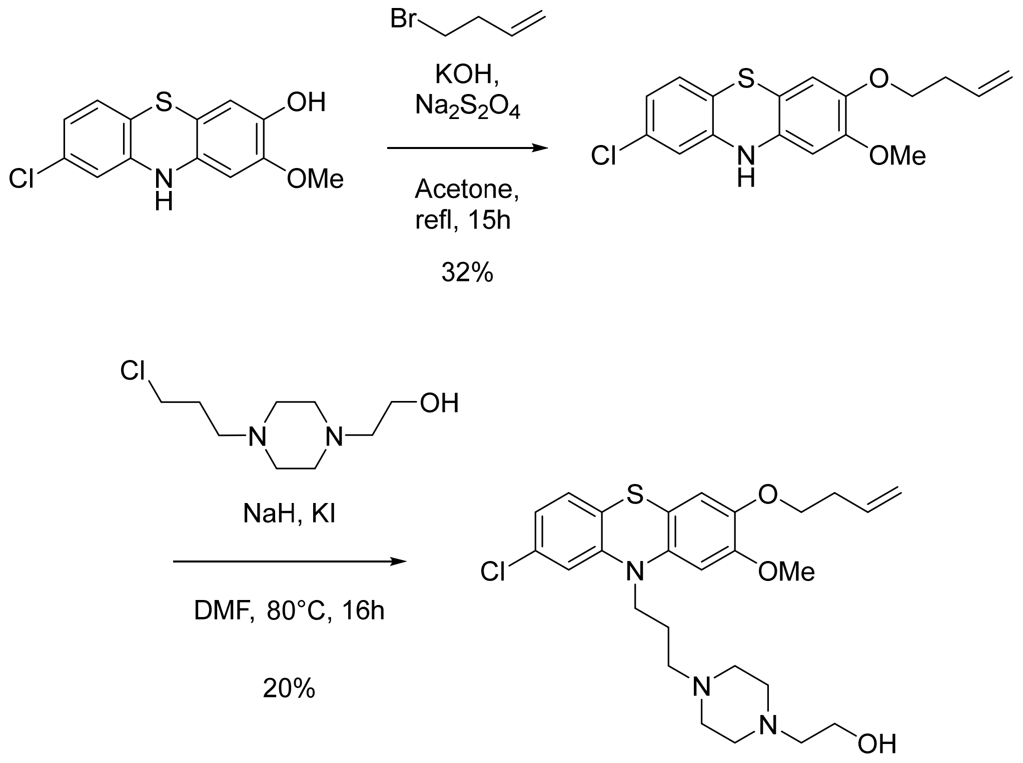

Scheme 7: Synthesis of the cmp9.

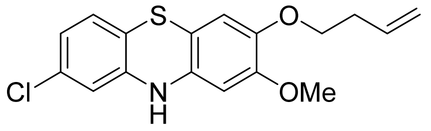

7-(But-3-en-1-yloxy)-2-chloro-8-methoxy-10*H*-phenothiazine

7-(But-3-en-1-yloxy)-2-chloro-8-methoxy-10*H*-phenothiazine was prepared from 2-chloro-8-methoxy-10*H*-phenothiazin-7-ol (276 mg, 0.99 mmol) using 4-bromobut-1-ene according to *General Procedure 1* to give the title compound (104 mg, 32%) as a grey solid.

^{1}H NMR (500 MHz, DMSO-d_6) δ 8.58 (s, 1H), 6.92 (d, *J* = 8.2 Hz, 1H), 6.76 (dd, *J* = 8.2, 2.1 Hz, 1H), 6.66 (d, *J* = 2.2 Hz, 1H), 6.60 (s, 1H), 6.38 (s, 1H), 5.85 (ddt, *J* = 17.0, 10.3, 6.6 Hz, 1H), 5.14 (dq, *J* = 17.3, 1.7 Hz, 1H), 5.05 (ddt, *J* = 10.3, 2.2, 1.2 Hz, 1H), 3.88 (t, *J* = 6.7 Hz, 2H), 3.70 (s, 3H), 2.40 (qt, *J* = 6.7, 1.5 Hz, 2H).
^{13}C NMR (126 MHz, DMSO) δ 149.47, 144.65, 143.99, 135.79, 135.39, 132.07, 127.90, 121.39, 117.36, 116.29, 113.97, 113.23, 105.79, 100.95, 68.88, 56.02, 33.68.
HRMS (+ESI) for $C_{17}H_{16}ClNO_2S$: $[M + H]^+$ calculated, 334.0669; found, 334.0661.

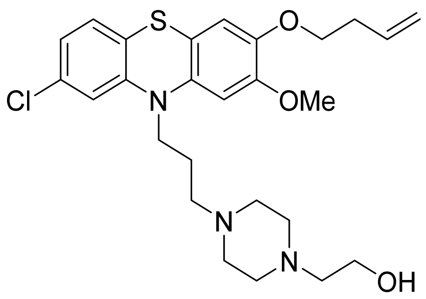

7-(But-3-en-1-yloxy)-2-chloro-8-methoxy-10-(3-(1-(2-hydroxyethyl)-4-piperazinyl)-propyl)phenothiazine

7-(But-3-en-1-yloxy)-2-chloro-8-methoxy-10-(3-(1-(2-hydroxyethyl)-4-piperazinyl)-propyl)phenothiazine was prepared from 7-(but-3-en-1-yloxy)-2-chloro-8-methoxy-10*H*-phenothiazine (99 mg, 0.30 mmol) according to *General Procedure 2* to give the title compound (30 mg, 20%) as a yellow oil.

^{1}H NMR (500 MHz, Chloroform-*d*) δ 7.02 (d, *J* = 8.1 Hz, 1H), 6.88 (dd, *J* = 8.2, 2.0 Hz, 1H), 6.83 (d, *J* = 2.0 Hz, 1H), 6.69 (s, 1H), 6.50 (s, 1H), 5.88 (ddt, *J* = 17.1, 10.3, 6.7 Hz, 1H), 5.16 (dq, *J* = 17.1, 1.6 Hz, 1H), 5.10 (dq, *J* = 10.2, 1.3 Hz, 1H), 3.98 (t, *J* = 7.0 Hz, 2H), 3.90 (t, *J* = 6.5 Hz, 2H), 3.84 (s, 3H), 3.73 – 3.69 (m, 2H), 2.82 – 2.50 (m, 14H), 1.98 (t, *J* = 6.9 Hz, 2H).
^{13}C NMR (126 MHz, $CDCl_3$) δ 149.37, 146.98, 144.67, 138.39, 134.14, 133.13, 127.96, 124.34, 122.24, 117.25, 115.98, 115.49, 113.35, 102.46, 69.02, 59.74, 57.16, 56.71, 54.91, 52.56, 51.97, 45.20, 33.64, 24.00.
HRMS (+ESI) for $C_{26}H_{34}ClN_3O_3S$: $[M + H]^+$ calculated, 504.2082; found, 504.2086.

Supplementary References

1 Jagtap, P. K. *et al.* Rational Design of Cyclic Peptide Inhibitors of U2AF Homology Motif (UHM) Domains To Modulate Pre-mRNA Splicing. *J Med Chem* **59**, 10190-10197, doi:10.1021/acs.jmedchem.6b01118 (2016).

2 Nodiff, E. A. *et al.* Synthesis of possible metabolites of chlorpromazine. III. 7,8-Disubstituted chlorpromazine derivatives. *Journal of Heterocyclic Chemistry* **7**, 203-208, doi:10.1002/jhet.5570070133 (1970).

3 ZHURAVLEV, S., ERMAKOVA, Z. & GRITSENKO, A. SYNTHESIS IN PHENOTHIAZINE SERIES. 9. SYNTHESIS OF 10-(GAMMA-[4-BETA-HYDROXYETHYL)-PIPERAZIN-1-YL]-PROPYL)-PHENOTHIAZINE AND ITS 2-CHLORO-, 2-ACETYL-, AND 2-PROPIONYL-SUBSTITUTED ANALOGS. *JOURNAL OF GENERAL CHEMISTRY USSR* **32**, 2211-& (1962).
