# Supplementary material for: Identification of phenothiazine derivatives as UHM-binding inhibitors of early spliceosome assembly
Source: Nat Commun. 2020 Nov 6;11:5621. doi: 10.1038/s41467-020-19514-1 (PMC7648758; doi:10.1038/s41467-020-19514-1)
Supplement: Supplementary file 3 — Reporting summary [file 41467_2020_19514_MOESM3_ESM.pdf]

nature research

Corresponding author(s): Thorsten Bach, Michael Sattler

Last updated by author(s): Sep 28, 2020

Reporting Summary

Nature Research wishes to improve the reproducibility of the work that we publish. This form provides structure for consistency and transparency in reporting. For further information on Nature Research policies, see our Editorial Policies and the Editorial Policy Checklist.

Statistics

For all statistical analyses, confirm that the following items are present in the figure legend, table legend, main text, or Methods section.

n/a	Confirmed	
☐	☒	The exact sample size (*n*) for each experimental group/condition, given as a discrete number and unit of measurement
☒	☐	A statement on whether measurements were taken from distinct samples or whether the same sample was measured repeatedly
☒	☐	The statistical test(s) used AND whether they are one- or two-sided<br>*Only common tests should be described solely by name; describe more complex techniques in the Methods section.*
☒	☐	A description of all covariates tested
☒	☐	A description of any assumptions or corrections, such as tests of normality and adjustment for multiple comparisons
☐	☒	A full description of the statistical parameters including central tendency (e.g. means) or other basic estimates (e.g. regression coefficient) AND variation (e.g. standard deviation) or associated estimates of uncertainty (e.g. confidence intervals)
☒	☐	For null hypothesis testing, the test statistic (e.g. *F*, *t*, *r*) with confidence intervals, effect sizes, degrees of freedom and *P* value noted<br>*Give P values as exact values whenever suitable.*
☒	☐	For Bayesian analysis, information on the choice of priors and Markov chain Monte Carlo settings
☒	☐	For hierarchical and complex designs, identification of the appropriate level for tests and full reporting of outcomes
☒	☐	Estimates of effect sizes (e.g. Cohen's *d*, Pearson's *r*), indicating how they were calculated

Our web collection on statistics for biologists contains articles on many of the points above.

Software and code

Policy information about availability of computer code

Data collection	Topspin v3.2 (Bruker)
Data analysis	Software used: XDS (Version March 1, 2015), Phenix (Version: 1.15.2), Coot (0.8.9), Pymol (v1.8.6), NMRPipe (Version 1.7), CCPN Analysis (V2), OriginPro (9.0), GraphPad Prism 6, Phaser (version 2.7.14), Molprobity programs (cluster 1999, clashlistcluster 1999 , mage Version 6.35.040409, prekin Version 6.35.040406, reduce Version 2.14, probe Version 2.6)

For manuscripts utilizing custom algorithms or software that are central to the research but not yet described in published literature, software must be made available to editors and reviewers. We strongly encourage code deposition in a community repository (e.g. GitHub). See the Nature Research guidelines for submitting code & software for further information.

Data

Policy information about availability of data

All manuscripts must include a data availability statement. This statement should provide the following information, where applicable:

- Accession codes, unique identifiers, or web links for publicly available datasets
- A list of figures that have associated raw data
- A description of any restrictions on data availability

Coordinates and structure factors for the PUF60 UHM domain in complex with 7,8 dimethoxyperphenazine have been deposited in the PDB with accession number 6SLO. NMR restraints for the SPF45 UHM domain in complex with 7,8-dimethoxyperphenazine has been deposited in the BMRB with accession code 50299

April 2020

Field-specific reporting

Please select the one below that is the best fit for your research. If you are not sure, read the appropriate sections before making your selection.

- [x] Life sciences
- [ ] Behavioural & social sciences
- [ ] Ecological, evolutionary & environmental sciences

For a reference copy of the document with all sections, see nature.com/documents/nr-reporting-summary-flat.pdf

Life sciences study design

All studies must disclose on these points even when the disclosure is negative.

Sample size	Sample sizes for dose response curves were chosen such that the inhibition curves showed saturation at low and high dose of inhibitor. No statistical methods were used to predetermine the sample size.
Data exclusions	No data were excluded from analysis.
Replication	Reproducibility of the binding of the compounds to the UHM domains were confirmed using complementary techniques such as NMR, FP assays and Alpha screen assays. In case of in vitro splicing, the assays were either carried out in duplicates or triplicates or the conclusion drawn were confirmed via a totally independent experiment using gradient centrifugation.<br>In case of in vitro splicing, all attempts at replication were successful. NMR experiments were carried out once. Chemical synthesis of all of the compounds was carried out once and the identities of the compounds were confirmed by NMR and mass spectrometry. FP assay for the binding of the cyclic peptide was carried out in triplicate and the FP assays to screen the library of the compounds were performed in duplicate.
Randomization	There was no allocation of groups done in this study and so no Randomization was required.
Blinding	There was no allocation of the groups done in this study and so no blinding was required.

Reporting for specific materials, systems and methods

We require information from authors about some types of materials, experimental systems and methods used in many studies. Here, indicate whether each material, system or method listed is relevant to your study. If you are not sure if a list item applies to your research, read the appropriate section before selecting a response.

Materials & experimental systems

n/a	Involved in the study	
[x]	[ ]	Antibodies
[x]	[ ]	Eukaryotic cell lines
[x]	[ ]	Palaeontology and archaeology
[x]	[ ]	Animals and other organisms
[x]	[ ]	Human research participants
[x]	[ ]	Clinical data
[x]	[ ]	Dual use research of concern

Methods

n/a	Involved in the study	
[x]	[ ]	ChIP-seq
[x]	[ ]	Flow cytometry
[x]	[ ]	MRI-based neuroimaging
